# Endogenous Hyaluronan Promotes Intestinal Homeostasis and Protects against Murine Necrotizing Enterocolitis

**DOI:** 10.3390/cells13141179

**Published:** 2024-07-11

**Authors:** Jeffery V. Eckert, Karni S. Moshal, Kathryn Burge, Adam Wilson, Hala Chaaban

**Affiliations:** Department of Pediatrics, Division of Neonatal-Perinatal Medicine, University of Oklahoma Health Sciences Center, Oklahoma City, OK 73104, USA; jeffrey-eckert@ouhsc.edu (J.V.E.); karni-moshal@ouhsc.edu (K.S.M.); kathryn-burge@ouhsc.edu (K.B.); adam-wilson@ouhsc.edu (A.W.)

**Keywords:** necrotizing enterocolitis (NEC), premature infants, hyaluronan (HA), intestinal development, crypt fission, NEC models

## Abstract

Necrotizing enterocolitis (NEC) is a complex, multifactorial gastrointestinal disorder predominantly affecting preterm infants. The pathogenesis of this condition involves a complex interplay between intestinal barrier dysfunction, microbial dysbiosis, and an altered immune response. This study investigates the potential role of endogenous hyaluronan (HA) in both the early phases of intestinal development and in the context of NEC-like intestinal injury. We treated neonatal CD-1 mouse pups with PEP1, a peptide inhibiting HA receptor interactions, from postnatal days 8 to 12. We evaluated postnatal intestinal developmental indicators, such as villi length, crypt depth, epithelial cell proliferation, crypt fission, and differentiation of goblet and Paneth cells, in PEP1-treated animals compared with those treated with scrambled peptide. PEP1 treatment significantly impaired intestinal development, as evidenced by reductions in villi length, crypt depth, and epithelial cell proliferation, along with a decrease in crypt fission activity. These deficits in PEP1-treated animals correlated with increased susceptibility to NEC-like injuries, including higher mortality rates, and worsened histological intestinal injury. These findings highlight the role of endogenous HA in supporting intestinal development and protecting against NEC.

## 1. Introduction

Necrotizing enterocolitis (NEC) is a devastating gastrointestinal disease primarily affecting premature infants [1]. NEC is marked by severe inflammation and necrosis of the small intestine, leading to sepsis, bowel perforation, and in severe cases, death [2,3,4]. The multifactorial etiology of NEC includes immature intestinal epithelium, bacterial dysbiosis, and compromised gut barrier function [3,5]. The vulnerability of the premature gut largely stems from its developmental immaturity. Studies indicate that key intestinal cellular components, such as Paneth cells, which are crucial for defending against pathogens and supporting stem cells, are less numerous in preterm infants [3,6,7,8,9]. Additionally, the production of mucins by goblet cells is diminished in preterm compared with term infants. This functional underdevelopment increases the risk of pathogen invasion and epithelial translocation [9,10,11]. Given these, and other, developmental susceptibilities of preterm infants, understanding factors that promote intestinal maturity and defenses is crucial to protect against NEC.

Hyaluronan (HA), a large negatively charged glycosaminoglycan, plays a significant role in intestinal development and protection against gastrointestinal inflammation and injury [12,13,14]. HA is considered a key element of the extracellular matrix, contributing to diverse biological functions including tissue hydration, cell adhesion, and modulation of inflammatory processes [15,16,17,18,19]. The biological effects of HA are contingent upon molecular size, with high molecular weight (HMW) HA molecules typically fostering tissue homeostasis and smaller HA fragments often associated with inflammatory immune responses [20,21,22,23].

The developmental and immunomodulatory effects induced by HA are mediated via the binding of specific receptors, including CD44 and toll-like receptors 2 and 4 (TLR2/4) [13,14]. In the intestine, HA is produced as HMW molecules and deposited on the basal surface of crypt epithelial cells. Pericryptal macrophage TLR4 is thought to bind HMW HA, inducing intestinal lengthening via crypt fission and expansion of LGR5^+^ (leucine-rich repeat-containing G protein-coupled receptor 5) intestinal stem cells. Previous studies have demonstrated the use of PEP1, a synthetic, 12-mer peptide that blocks HA interactions with its receptors [24], significantly and negatively impacts intestinal development and susceptibility to dextran sodium sulfate (DSS) colitis and radiation injury models in mice [12,13,14,25]. 

Despite well-documented roles in gut development, the role of endogenous HA in the context of NEC remains unexplored. We hypothesized that inhibition of endogenous HA receptor-binding via PEP1 would lead to detrimental effects on intestinal morphology and increase the incidence and severity of NEC-like injury. The goal of this study was to investigate the role of endogenous HA in the early stages of intestinal development in neonatal mice and the potential vulnerability to NEC-like intestinal injury associated with disruption of endogenous HA signaling in the immature gut.

## 2. Materials and Methods

### 2.1. Animal Experiments

All animal experiments and procedures were conducted in accordance with guidelines approved by the University of Oklahoma Health Sciences Center Institutional Animal Care and Use Committee (reference #22-035-EFHI). Timed pregnant CD-1 mouse dams (Charles River Laboratories, Wilmington, MA, USA) were housed individually, maintained under a 12 h light/dark cycle, and provided ad libitum access to food and water. Pups of both sexes were randomized at P6 into two groups: PEP1 (peptide inhibitor of endogenous HA receptor-binding; H2N-GAHWQFNALTVR-OH) or control (scrambled peptide; H2N-WRHGEALTAVNQ-OH) [14]. Peptides were purchased from Thermo Fisher Scientific (Carlsbad, CA, USA). Based on previous literature [12], PEP1 or scrambled peptide, dissolved in distilled water, was administered intraperitoneally (i.p.) at a dose of 15 mg/kg bodyweight every other day, from postnatal (P) day 8 (P8) to P12. Body weights were recorded in both groups, and pups were returned to dams following injections. An initial assessment for sex differences was conducted and found no significant differences in response to the treatments. Therefore, results from both sexes were combined for the final analysis.

### 2.2. NEC-like Intestinal Injury Model

To assess mouse pup susceptibility to NEC-like intestinal injury, we employed the dithizone/*Klebsiella* (DK) NEC model [26]. This model involves disrupting Paneth cells with dithizone followed by an oral bacterial challenge to simulate NEC conditions. Specifically, P14 pups received an i.p. injection of dithizone (33 mg/kg) diluted in ethanol/ammonium hydroxide, followed by an oral gavage of 1 × 10^8^ colony forming units (CFU) of *Klebsiella pneumoniae*/kg (ATCC, Manassas, VA, USA; stock#10031). Based on prior experiments evaluating the impact of exogenous HA on histological injury scores in an NEC model, a minimum of 8 animals per group was determined to be necessary to achieve 80% power for detecting a significant difference. Considering a mortality rate of 40–50% in previous studies, we increased the sample size to include at least 16 animals per group. 

Within this experimental framework, mouse pups originally treated with either PEP1 or a scrambled peptide were further randomized, resulting in four distinct experimental groups (Supplementary Figure S1): (1) Control (*n* = 16): i.p. injections every other day of scrambled peptide (control) from P8 to P12, followed by normal saline (NS) i.p., and culture media gavage; (2) PEP1 (*n* = 16): i.p. injections every other day of PEP1 from P8 to P12, followed by NS i.p. and culture media gavage; (3) NEC + scramble (*n* = 17): i.p. injections every other day of scrambled peptide (control) from P8 to P12, with dithizone i.p. and *Klebsiella* oral gavage on P14; (4) NEC + PEP1 (*n* = 18): i.p. injections of PEP1 every other day from P8 to P12, with dithizone i.p. and *Klebsiella* oral gavage on P14. On the day of the experiment, pups were removed from the dam and provided thermal support in a 33 °C incubator. Pups were monitored for a total of 16 h for clinical illness and survival. At the study’s conclusion, surviving pups were anesthetized with isoflurane and euthanized via bilateral thoracotomy and cardiac puncture. Blood and tissue were collected and flash-frozen in liquid N_2_ for downstream analyses. Sections from terminal ileal tissue were harvested in RNAlater (Invitrogen, Carlsbad, CA, USA) for gene expression analysis, or fixed in either 10% buffered formalin or Carnoy’s solution (60% ethanol, 30% chloroform, 10% glacial acetic acid), as appropriate, for histology and immunohistochemical staining.

### 2.3. Histology for Quantification of Villus Height, Crypt Dept, Crypt Fission, Paneth, and Goblet Cell Numbers

Serial histological sections of 5 µm thickness were cut, deparaffinized, rehydrated, and stained with hematoxylin and eosin (H&E) for analysis. For quantitative assessment, crypts were counted from well-oriented sections where the bilateral crypt–villus junctions were intact. Villus measurements were taken only from sections in which the central lymphatic channel was visible, extending from the villus base to the tip. Utilizing ImageJ software (version 1.54k; National Institutes of Health, Bethesda, MD, USA), at least 200 villus heights and crypt depths were measured per animal, with measurements performed on 8 animals per group [10,27]. Crypt fission frequency was assessed by examining histological sections for crypt bifurcation, which results in at least two flask-shaped bases sharing a common crypt–villus junction [27,28]. For determination of Paneth and goblet cell numbers, sections were stained with Periodic Acid Schiff (PAS)/Alcian blue (AB) (Millipore Sigma, St. Louis, MO, USA), as previously described [9]. 

### 2.4. Intestinal Injury Scoring

The severity of the histological injury was assessed from sections of the terminal ileum of surviving pups by 2 independent investigators using a five-point scoring system for NEC-like intestinal injury, as previously described [26,29]: Grade 0: intact mucosal morphology; Grade 1: loss of epithelial cells at tip of villus; Grade 2; mid-villus sloughing; Grade 3: mucosal necrosis with loss of villi; Grade 4: transmural necrosis. Scores were based on the highest score observed on three to five sections within the same sample. A score of 2 or more was designated as NEC.

### 2.5. Immunohistochemistry and Immunofluorescence

Paraffin-embedded sections were deparaffinized, rehydrated, and steamed for 30 min in citrate buffer for antigen retrieval. Sections were blocked with 5% goat serum in Tris-buffered saline with 0.05% Tween-20, and incubated with primary antibody (Ki-67; 1:100; Thermo Fisher Scientific). Horseradish peroxidase-conjugated secondary antibodies (rabbit anti-goat IgG 1:200 [Abcam, Waltham, MA, USA]; goat anti-rabbit IgG, 1:100, Abcam) and Signal Stain DAB Substrate Kit (Cell Signaling Technology, Danvers, MA, USA) were used for detection, with hematoxylin for nuclear counterstaining. Sections were processed for antigen retrieval using citrate-based unmasking solution (Vector Laboratories, Newark, CA, USA), microwaved at 800 W for 5 min, cooled to 55 °C, and microwaved again for 5 min. After cooling to room temperature, sections were permeabilized with phosphate-buffered saline (PBS)/Triton X-100-0.25%/0.2% gelatin and blocked with 5% bovine serum albumin (BSA) in a humidified chamber. Overnight staining at 4 °C was performed with biotin–hyaluronic acid-binding protein (HABP) antibody (AMS.HKD-BC41; AMSBIO, Cambridge, MA, USA) at 1:75 in PBS with 1% BSA, followed by secondary and nuclear staining with anti-Alexa 488 (1:400; S11223; Thermo Fisher Scientific) and DAPI (4′,6′-diamidino-2-phenylindole; D9542; Sigma-Aldrich, St. Louis, MO, USA), respectively. After washing, sections were mounted using Shandon^TM^ Immu-Mount^TM^ (Thermo Fisher Scientific) and imaged using an SP8 confocal microscope with LASX software (version 3.7.2; Leica, Teaneck, NJ, USA). Photomicrographs were analyzed using Fiji/ImageJ (NIH), with corrected total cell fluorescence (CTCF) calculated for immunofluorescence intensity comparison across the tissue.

### 2.6. Plasma Cytokines

Mouse plasma samples were diluted 1:2 for cytokine analysis using the Simple Plex^TM^ Ella system (ProteinSimple, Bio-Techne, Oxford, UK) [30]. Quality control (QC) samples were prepared with reconstituted lyophilized recombinant protein standards, per the manufacturer’s instructions. Sample information and dilution factors were input using Simple Plex Runner software (version 4.1.0.22; ProteinSimple, Bio-Techne, Minneapolis, MN, USA). Results for each cytokine were automatically displayed in triplicate. Raw signal levels were reported in relative fluorescence units (RFU), and calculations provided mean RFU values, standard deviation, and coefficient of variance (CV). Final cytokine concentrations in pg/mL were determined by back-fitting RFU values to the standard curves, considering the dilution factors.

### 2.7. Statistical Analysis

Statistical analysis was conducted using Prism v10.1.2 (GraphPad, San Diego, CA, USA). Data are presented as mean ± standard deviation (SD) or standard error of the mean (SEM), as appropriate. Differences between the two groups were analyzed using Student’s *t*-tests. Multiple groups were analyzed by one-way or two-way analysis of variance (ANOVA) with post hoc Tukey, Mann–Whitney U, or Kruskal–Wallis tests, as appropriate. A *p*-value of less than 0.05 was considered statistically significant. 

## 3. Results

### 3.1. Effect of PEP1 on Bodyweight and Intestinal Morphology

**Figure 1:**
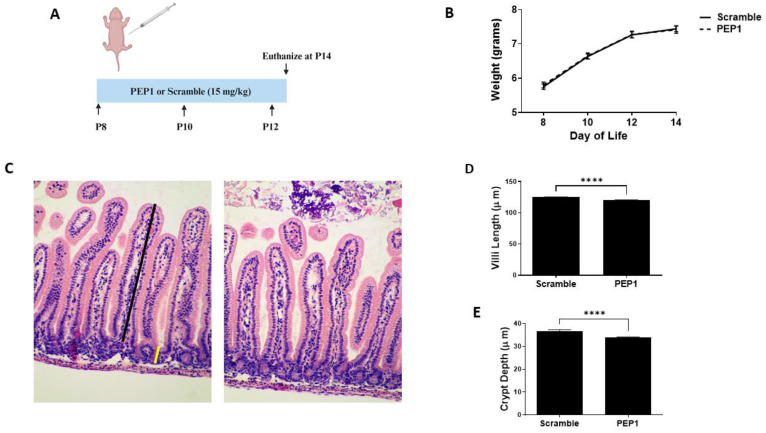
Growth and intestinal morphology in PEP1-treated mice versus Scrambled Peptide: (**A**) PEP1 or scrambled peptide (scramble) was given intraperitoneally at 15 mg/kg every other day from P8 to P12. (**B**) Growth curve representing weights of pups in both groups (*n* = 34 PEP1, *n* = 33 scramble). (**C**) Representative histological sections stained with hematoxylin and eosin from PEP1 and scramble group. Black line represents length measurement for villi, yellow line crypt depth measurement. Quantification of villi length (**D**) and crypt depth (**E**) for both groups (*n* > 200 crypts or villi from 8 pups per group; **** *p* < 0.0001). Data presented as mean ± SEM.

Previous studies show endogenous HA influences growth and intestinal morphology when administered from three to eight weeks post-birth [12]. Here, we sought to investigate the effects of PEP1 administration from P8 to P12 (Figure 1A), a developmental period corresponding with preterm human intestinal development at 22–24 weeks gestation and increased susceptibility to NEC [31]. Mouse pups in both the PEP1-treated and the control group followed a similar growth trajectory, as illustrated in Figure 1B, indicating short-term PEP1 administration did not influence overall growth. While gross histological analysis failed to indicate significant changes between PEP1 or scrambled peptide treatments (Figure 1C), notable distinctions between the groups were observed with intestinal morphology, particularly villus height and crypt depth. Ileal sections from pups treated with PEP1 displayed shorter villi, with an average length of 120.3 ± 0.15 µm compared with 125.2 ± 0.08 µm in the control group (Figure 1D; *p* < 0.0001). Furthermore, crypt depths in the PEP1-treated group were significantly shallower, measuring 33.99 ± 0.22 µm, versus 36.62 ± 0.19 µm in the control group (Figure 1E; *p* < 0.0001). These findings confirm endogenous HA affects early postnatal gut development.

**Figure 2:**
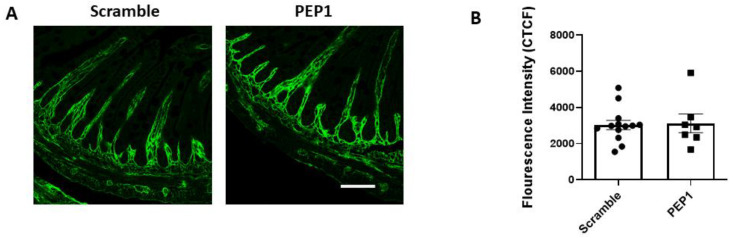
HA-binding protein (HABP) immunofluorescence in P14 PEP1- and scramble-treated mouse pup terminal ileum: (**A**) Representative images illustrating HA localization in the terminal ileum via binding of HABP (green). (**B**) Fluorescence intensity of scramble- and PEP1-treated pup HABP staining. Data presented as mean ± SEM. Scale bar = 50 µm.

Next, we examined whether PEP-1 treatment influences the distribution of HA within the intestinal tissue of mouse pups. Terminal ileal sections were immunostained for HA using a hyaluronic acid-binding protein (HABP), revealing that HA consistently localized in the lamina propria adjacent to the crypts and villi in both the scramble and PEP1-treated groups (Figure 2A). No differences were observed in the pattern or intensity of HA staining (Figure 2B) between the two groups, suggesting PEP1 does not alter the distribution or overall presence of HA in the tissue. This observation is consistent with previous reports indicating that PEP1 does not impact HA synthesis or deposition in the gut [24,28].

### 3.2. Effect of PEP1 on Intestinal Epithelial Proliferation, Differentiation, and Crypt Fission

**Figure 3:**
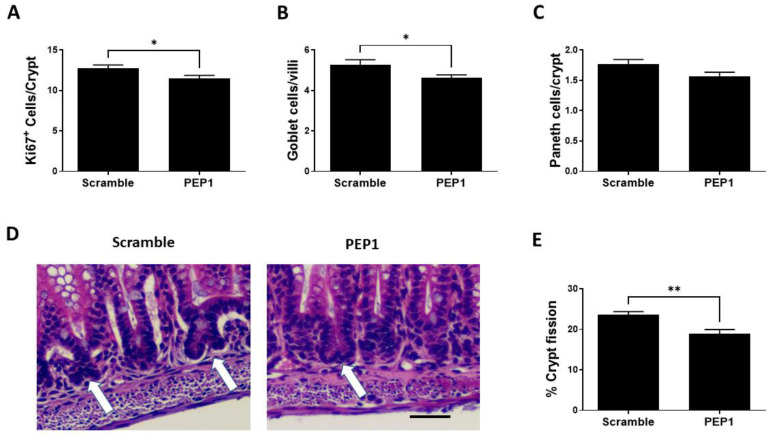
Effect of PEP1 on intestinal epithelial proliferation, differentiated cells, and crypt fission: (**A**) Quantification of Ki67-positive cells per crypt showing a significant reduction in cell proliferation in PEP1-treated versus control animals (*n* > 50 crypts/group from at least 3 animals). (**B**) Quantification of goblet cells per villi, indicating a significant decrease in the number of goblet cells in PEP1-treated pups (*n* > 50 villi/group from at least 3 animals). (**C**) No significant difference in the number of Paneth cells per crypt between groups (*n* > 50 crypts/group from at least 3 animals). (**D**) Representative histological sections of intestinal tissue for crypt fission (white arrows). Scale bar represents 50 µm. (**E**) Percentage of crypt fission, with a significant decrease observed in the PEP1 group. Data presented as mean ± SEM, * *p* < 0.05, ** *p* < 0.01.

Next, we sought to determine the effect of PEP1 on intestinal epithelial proliferative capacity, cell differentiation, and intestinal lengthening. Epithelial proliferation is commonly assessed by expression of the proliferative marker, Ki-67, and PEP1 administration during early intestinal development was associated with a significant decrease in Ki67-positive cells per crypt compared to scrambled controls (11.46 ± 0.41 vs. 12.72 ± 0.43 respectively; *p* = 0.039; Figure 3A). In addition, the number of goblet cells was significantly lower in the PEP1 group compared with that of control (4.65 ± 0.15 vs. 5.25 ± 0.26 cells per villus, respectively; *p* = 0.035; Figure 3B). No significant differences were observed between PEP1 and control groups in the number of Paneth cells per crypt (1.57 ± 0.06 vs. 1.76 ± 0.07 cells per crypt, respectively; *p* = 0.057; Figure 3C). Further analysis showed approximately a 15% reduction in crypt fission per field in the PEP1 group relative to the scrambled controls (19% vs. 22.5% per field, respectively; *p* = 0.0364; Figure 3D,E). 

### 3.3. Effect of PEP1 on NEC-like Intestinal Injury in Mouse Pups

**Figure 4:**
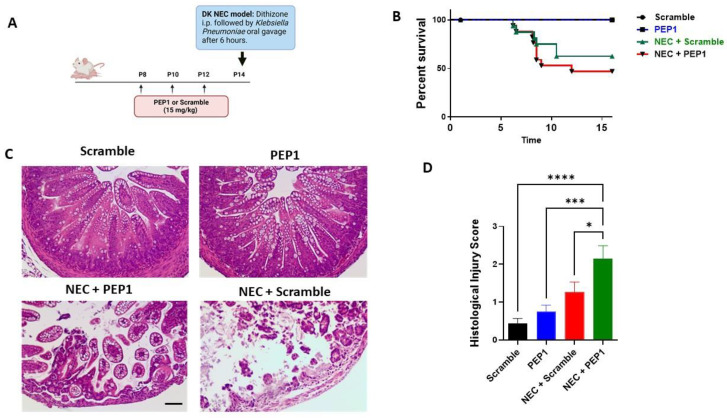
Treatment impacts NEC-like injury and survival in neonatal mice: (**A**) Experimental design illustrating the timeline for intraperitoneal (i.p.) administration of either PEP1 or a scramble peptide (15 mg/kg, P8 to P12), as well as NEC induction (dithizone and *Klebsiella pneumoniae* at P14). The dithizone/*Klebsiella* (DK) NEC model induction at P14 with dithizone i.p. followed by oral gavage of *Klebsiella pneumoniae* after 6 h. (**B**) Kaplan–Meier survival curves showing percentage survival over time across all groups: control (*n* = 16), PEP1 (*n* = 16), NEC + scramble (*n* = 17), and NEC + PEP1 (*n* = 18). (**C**) Representative hematoxylin and eosin-stained ileal sections from each group, displaying intestinal architecture at 20× magnification. (**D**) Histological injury scores of sections from terminal ileum of the surviving pups from each group. Data presented as mean ± SEM, * *p* < 0.05, *** *p* < 0.001, **** *p* < 0.0001.

To elucidate the role of PEP1 in NEC susceptibility, pups were randomized into four experimental groups (Supplementary Figure S1): control, PEP1, NEC + scramble, and NEC + PEP1. Scramble control and PEP1 groups received intraperitoneal (i.p.) injections every other day from P8 to P12 of a scrambled peptide or PEP1 (15 mg/kg), respectively. On P14, pups received an i.p. injection of dithizone or vehicle, followed six hours later by oral gavage with *Klebsiella pneumoniae* or culture media (Figure 4A). Survival analysis revealed a trend toward increased susceptibility to NEC in the NEC + PEP1 group versus NEC + scramble (69% vs. 53.3%, respectively; Figure 4B) but did not reach statistical significance. Histological assessment of the ileum revealed normal intestinal morphology in both scramble control and PEP1 groups. In contrast, the NEC + scramble and NEC + PEP1 groups exhibited significant intestinal damage, characterized by vacuolation, mucosal edema, and loss of villus architecture (Figure 4C). Notably, the NEC + PEP1 group displayed higher histological injury scores than the NEC + scramble (2.154 ± 0.33 vs. 1.267 ± 0.2667; *p* = 0.0492; Figure 4D). Moreover, the prevalence of NEC, defined as a histological injury score of two or above, was significantly higher in the NEC + PEP1 group compared to the NEC + scramble group (76.9% vs. 53.3%; *p <* 0.001). 

**Figure 5:**
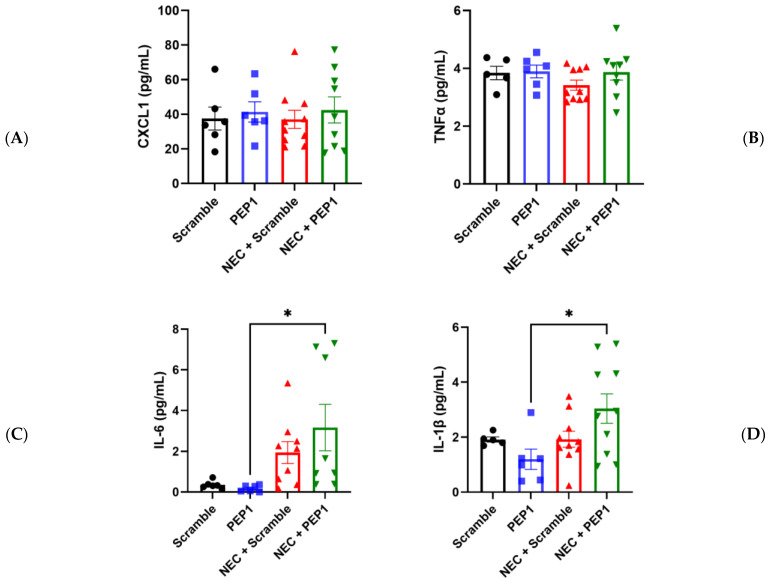
Effect of PEP1 on Serum Cytokine Levels of IL-6 and IL-1β. Serum concentrations of (**A**) CXCL1, (**B**) TNFα, (**C**) IL-6, and (**D**) IL-1β from at least 6 pups from the experimental groups (*n* = 6, scramble control; *n* = 6 PEP1; *n* = 9, NEC + scramble; *n* = 8, NEC + PEP1). The NEC + PEP1 group shows a significant increase in IL-6 and IL-1β levels compared with PEP1 alone. Data presented as mean ± SEM, * *p* < 0.05. Statistical comparison was performed using one-way ANOVA with Tukey’s multiple comparisons test.

In partial support of the above findings, pups in the NEC + PEP1 group trended toward higher serum CXCL1 and TNF-α values compared with all other groups (Figure 5A–C). Significant differences were only indicated for NEC + PEP1 compared with PEP1 in IL-6 (3.165 ± 1.136 vs. 0.1763 ± 0.0646 pg/mL, * *p* < 0.0395; Figure 5C) and IL-1β (3.038 ± 0.5369 vs. 1.196 ± 0.3703 pg/mL, * *p* < 0.0264; Figure 5D). Collectively, these findings suggest that PEP1 may potentiate the severity of NEC, highlighting the modulatory role of endogenous HA in intestinal inflammation.

## 4. Discussion

NEC is a severe gastrointestinal disorder primarily affecting premature infants, with current treatments often limited to supportive care, broad-spectrum antibiotics, and surgical intervention in 30% of cases [32]. The long-term complications of NEC, such as neurodevelopmental delays and short bowel syndrome, underscore the critical need for advancing our understanding of this devastating disease and develop strategies to promote postnatal intestinal maturation [33,34].

In this study, we investigated the effects of PEP1, an inhibitor of HA-receptor binding [12,13,14,25], on early gut development and susceptibility to an NEC-like intestinal injury model. Administration of PEP1 from P8 to P12 led to significant alterations in intestinal morphology, notably shorter villi, shallower crypts, and reduced numbers of Ki67-positive proliferative cells and goblet cells, without significantly affecting Paneth cell counts. The observed decrease in crypt fission further suggests that endogenous HA is crucial for intestinal growth and remodeling.

The biological role of HA is influenced by the MW and environmental context [9]. In general, HMW HA synthesized by hyaluronan synthases (HASs), enhances tissue hydration, acts as an antioxidant, and inhibits endothelial cell growth by physically limiting extracellular matrix space. In contrast, oligo-HA (<10 kDa), low molecular weight (LMW) HA (10–250 kDa), and medium molecular weight (MMW) HA (250–1000 kDa), typically produced through the endogenous fragmentation of larger HA polymers following tissue injury, induce inflammation, activate the innate immune system, stimulate growth factor production, and promote angiogenesis [12,19,35,36,37,38,39,40]. These lower MW HA fragments play significant roles in inflammatory and fibrotic diseases and contribute to wound healing and tissue repair [23]. HMW HA also facilitates wound healing by providing an initial scaffolding post-injury, supporting cell migration, angiogenesis, and tissue remodeling driven by lower MW HA fragments [19]. Additionally, HMW HA can promote CD44 receptor crosslinking, inducing anti-inflammatory cytokine synthesis and aiding wound inflammation resolution [21].

In the gut, the role of HA, whether endogenously produced or exogenously introduced, is well documented, with studies indicating its essential function in maintaining the extracellular matrix, promoting tissue hydration, and facilitating cell migration and proliferation [16,18,41]. Studies demonstrated that long-term i.p. administration of HMW HA (750 kDa) induces colonic epithelium proliferation in healthy mice and provides radioprotection by increasing crypt survival and reducing apoptosis [12]. Similarly, systemic administration of HA 750 kDa was shown to be protective against DSS-induced colitis [25]. 

In contrast, oral administration of exogenous lower MW HA, specifically HA 35 kDa, has shown protective effects. Hill et al. reported that oral HA 35 kDa induces beta defensin-2 expression through TLR-4, enhancing antimicrobial defenses [42]. HA 35 kDa also reduces the severity of bacterial infections, such as Citrobacter rodentium, by decreasing bacterial translocation and increasing tight junction protein ZO-1 expression [43]. Furthermore, we previously showed that oral HA 35 kDa promotes postnatal intestinal development, and protects against NEC by enhancing epithelial barrier defenses, reducing intestinal permeability, and lowering proinflammatory cytokine release [26,29]. These findings highlight that while systemic administration of HMW HA supports gut development, injury repair, and anti-inflammatory responses, oral administration of lower MW HA, specifically HA 35 kDa, is protective against intestinal inflammation and bacterial infections by strengthening epithelial barriers and innate defenses.

Previous studies utilizing PEP1, as a surrogate for the role of endogenous HA, have shown that prolonged treatment over five weeks in mice leads to significant reductions in the overall length of the intestine and colon, accompanied by decreases in Paneth cell and goblet cell densities, and crypt fission rates [12,14,16,28]. In addition, mouse pups treated with PEP1 from P7 to P14 showed a significant reduction in intestinal stem cell proliferation, crypt height, and villus depth. Riehl et al. demonstrated small intestinal and colonic epithelial proliferation, and apoptosis is dependent upon endogenous HA receptor binding of both CD44 and TLR4 in vivo [14]. A blockade of endogenous HA receptor binding utilizing PEP1 reduced Lgr5^+^ stem cell expansion in the crypts, decreased Paneth cell numbers, and led to a decline in crypt fission. 

Our results similarly showed that PEP1 treatment was associated with reductions in goblet cell numbers and proliferating cells, although no significant difference in intestinal length was noted, likely due to the shorter treatment duration. Importantly, the reduction in goblet cell numbers and cell proliferation is particularly relevant for NEC prevention, as increased susceptibility of the immature preterm infant intestine has been attributed, in part, to reduced goblet cells and the inability of the preterm intestinal epithelium to regenerate post-injury [2,44]. 

Our study also revealed a significant increase in susceptibility to NEC-like injuries upon inhibiting HA signaling, affirming its protective role in intestinal health. This aligns with previous studies reporting the importance of endogenous HA. For example, HA has been shown to protect against DSS-induced colitis by activating TLR4 and promoting the production of PGE2 (prostaglandin E2) [14]. Similarly, HA supplementation before radiation-induced injury increases crypt survival and confers protection, in part through TLR activation and COX2 (cyclooxygenase-2) production [13]. Although crypt fission has not been extensively studied in the context of NEC, it has been shown to play a role in regeneration post-injury. Future studies are needed to determine its relevance to NEC prevention.

Our study is subject to limitations. We focused on outcomes at P14 following a brief regimen of PEP1 treatment, which was selected for its relevance to the developmental stage of the preterm intestinal epithelium [31]. This timeframe, while critical, may affect broader outcomes such as overall weight and intestinal length. In addition, our analysis was restricted to the terminal ileum, which is of particular interest in NEC development; however, results may vary in different intestinal regions. 

We also utilized only one HA inhibitor, PEP1, in this study. While PEP1 is well documented for its specific effects on HA binding and signaling, other HA inhibitors, such as 4-methylumbelliferone (4-MU), which inhibit the synthesis of HA [45], may yield different findings or offer additional insights into HA’s role in NEC. 

Furthermore, we did not directly explore the mechanisms of action in this study. Parallel studies in murine models suggest that this could be in part due to the activation of TLR4 by HA, triggering a cascade involving COX-2 and PGE2, which culminates in the activation of the epithelial growth factor receptor (EGFR) [14,28,46,47].

## 5. Conclusions

In conclusion, our findings underscore the role of HA in early intestinal development and mitigating NEC-like injuries. These insights support the exploration of HA pathways as potential therapeutic targets to enhance intestinal resilience and prevent NEC in preterm infants. Future research should prioritize the identification of factors that influence HA production and degradation, along with strategies to augment HA synthesis. Moreover, studies should investigate methods to expedite intestinal maturation through HA, aiming to reduce the incidence of NEC. 

## Data Availability

The data presented in this study are available on request from the corresponding author.

## Supplementary Materials

The following supporting information can be downloaded at: https://www.mdpi.com/article/10.3390/cells13141179/s1, Figure S1: Experimental design for the DK NEC modelMice were divided into four groups: Scramble Control, PEP1, NEC + Scramble, and NEC + PEP1. From postnatal day (P) 8 to P12, mice received every other day intraperitoneal (i.p.) injections of either scrambled peptide or PEP1 (15 mg/kg). At P14, the NEC challenge was induced by intraperitoneal injection of dithizone followed by oral gavage with Klebsiella pneumoniae after 6 hours. Control groups received normal saline i.p. followed by gavage with culture media.
