# Supplementary material for: Endogenous Hyaluronan Promotes Intestinal Homeostasis and Protects against Murine Necrotizing Enterocolitis
Source: Cells. 2024 Jul 11;13(14):1179. doi: 10.3390/cells13141179 (PMC11274784; doi:10.3390/cells13141179)
Supplement: Supplementary file 1 [file cells-13-01179-s001.zip › cells-3062132-supplementary.pdf]

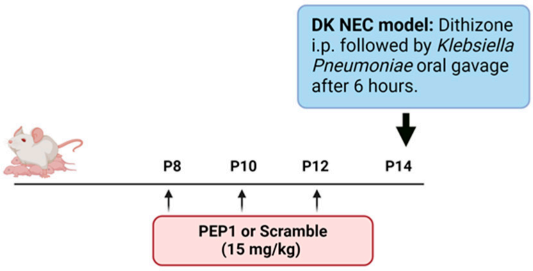

Group	P7- P14	NEC challenge at P14
**Scramble Control**	Scrambled Peptide *i.p.* daily	Normal saline *i.p.* followed by gavage culture media after 6 hours.
**PEP1**	PEP1 *i.p.* daily	Normal saline *i.p.* followed by gavage culture media after 6 hours.
**NEC + Scramble**	Scrambled Peptide *i.p.* daily	Dithizone *i.p.* followed by oral *Klebsiella pneumoniae* after 6 hours.
**NEC + PEP1**	PEP1 *i.p.* daily	Dithizone *i.p.* followed by oral *Klebsiella pneumoniae* after 6 hours.

Figure S1: Experimental design for the DK NEC modelMice were divided into four groups: Scramble Control, PEP1, NEC + Scramble, and NEC + PEP1. From postnatal day (P) 8 to P12, mice received every other day intraperitoneal (i.p.) injections of either scrambled peptide or PEP1 (15 mg/kg). At P14, the NEC challenge was induced by intraperitoneal injection of dithizone followed by oral gavage with Klebsiella pneumoniae after 6 hours. Control groups received normal saline i.p. followed by gavage with culture media.
